# Determination of productivity, yield and bioactivity of propolis extract produced by *Tetragonula* spp. Cultivated in Modular *tetragonula* hives

**DOI:** 10.1016/j.heliyon.2023.e17304

**Published:** 2023-06-17

**Authors:** Muhammad Yusuf Abduh, Fahmi Ramdhani, Albert Setiawan, Ghiffary Rifqialdi, Anasya Rahmawati, Ima Mulyama Zainudin

**Affiliations:** a School of Life Sciences and Technology, Institut Teknologi Bandung, Jalan Ganesha 10, 40132 Bandung, Indonesia; b University Center of Excellence for Nutraceuticals, Bioscience and Biotechnology Research Center, Institut Teknologi Bandung, Jalan Ganesha 10, 40132 Bandung, Indonesia

**Keywords:** Anti-microbial activity, Modular *tetragonula* hive, Propolis, *Pinus merkusii*, *Tetragonula* spp.

## Abstract

This study investigated the effects of microclimate conditions on the activity of *Tetragonula laeviceps*, *Tetragonula biroi*, and *Tetragonula drescheri* cultivated in Modular *Tetragonula* Hives for producing crude propolis. The hives were equipped with sensors that recorded microclimate data within the hive as well as the total activity of bees entering and leaving the hives. This study also investigated the effects of cultivating *T. laeviceps*, *T. drescheri*, and *T. biroi* with *P. merkusii* resin towards the productivity of crude propolis and the effects of different extraction methods on the yield, total phenolic content, total flavonoid content, and antibacterial activity of propolis extract produced by the *Tetragonula* spp. Based on the statistical analysis, there is a significant positive correlation between temperature and light intensity towards the activity *Tetragonula* spp. entering and leaving the beehives. The productivity of crude propolis lies in the range of 1.22–5.88 g/colony/week whereas the yield of propolis extract varies from 15.12 to 24.17%. The total phenolic and flavonoid content of the propolis extract lies in the range of 123.81–343.93 mg GAE/g and 5.48–35.77 mg QE/g, respectively. The highest propolis yield (32.45 ± 0.90%) was obtained from the crude propolis produced by *T. drescheri* followed by Soxhlet extraction method. Propolis extract with the highest phenolic content (343.93 ± 44.32 mg GAE/g) and flavonoid content (35.77 ± 9.94 mg QE/g) was obtained from the propolis produced by *T. laeviceps* followed by maceration method. All the propolis extracts inhibited the growth of *Staphylococcus aureus* with the inhibition diameter varies from 6.58 ± 0.04 mm to 9.70 ± 0.7 mm which be considered as moderate antimicrobial activity.

## 1 Introduction

Propolis is a substance produced by bees that are used in the construction of their hives as well as to cover gaps at the entrance of the hive; this protects them from both bacterial contamination and other predators [1,2]. Stingless bees (*Tetragonula* sp.) are reported to have a higher capacity for production of propolis as compared to honeybee species (*Apis* sp.). Many species of stingless bees are widely cultivated in Indonesia, including *Tetragonula laeviceps, Tetragonula drescheri, Tetragonula biroi, Tetragonula minor, Tetragonula fuscobalteata, Tetragonula clypearis,* and *Wallacetrigona incisa* [3]. *T. laeviceps* is the most widely distributed in the Indonesian archipelago and can be found on the islands of Sumatera, Java, Borneo, Sulawesi, and Ambon Island. Meanwhile, *T. drescheri* is present on the islands of Sumatera, Java, and Borneo, while *T. biroi* is widely distributed in Irian Jaya [3].

Local stingless beekeepers in Indonesia cultivate bees in hives made of tree trunks, bamboo, wooden boxes, or other hard materials such as acrylic. Typically, local beekeepers harvest the propolis by destroying the beehive, which disrupts the bee colony. This problem could be alleviated with the application of Modular *Tetragonula* Hive (MOTIVE), which allows beekeepers to harvest the propolis without needing to destroy the hive. MOTIVE has an innovative design that combines a wooden box hive with a detachable propolis frame that contains small holes; bees are incentivized to fill the holes with propolis, allowing propolis collection and production to be optimized. MOTIVE units have also been developed for use with instrumentation systems. By outfitting the beehive with these instrumentation systems, beekeepers can collect microclimate data, allowing them to monitor the health of the colony with respect to the microclimate conditions within the hive [4,5].

Propolis extracts can be obtained by using conventional extraction methods such as Soxhlet, maceration, or reflux extraction of the crude propolis. Several active compounds in propolis are flavonoids, aromatic acids, terpenoids, phenylpropanoids, and fatty acids [6]. Due to the presence of these active compounds, many studies have been conducted on the bioactivity of propolis, which can have antibacterial, antifungal, antiviral, anti-inflammatory, local anaesthetics, immuno-stimulant, antiparasitic, and anticancer properties. However, the biological activity of propolis is also influenced by the quantitative and qualitative composition of the phytochemicals in the propolis, which in turn depends on the local plant vegetation at the sites where the resin was collected [7].

*Pinus merkusii* has a high amount of resin content [8] and one of the most favoured sources of resin by stingless bees to produce propolis [9]. Various studies have reported that *P. merkusii* contains a relatively high amount of total phenolic content and total flavonoid content. According to Masendra et al. [10], total phenolic content and total flavonoid content *P. merkusii* bark are in the range of 421.4–486.5 mg GAE/g and 55.2–367.4 mg CE/g, respectively. In another study, Aloui et al. [11] reported that pine resin shows a promising bioactivity with an antioxidant activity (IC_50_) in the range of 15 ± 0.59 mg/mL to 17 ± 0.11 mg/mL and antibacterial capacity with an average inhibition zone diameter of 1.46–2.13 cm. As such, valorisation of pine resin as a source of resin for stingless bees to produce propolis may increase the bioactivity of the propolis extract.

At present, systematic studies that report the effects of microclimate conditions on the activity of different types of stingless bees in producing propolis are very scarce. In addition, there are no published articles that systematically investigate the influence of specific resin on the productivity of crude propolis and composition of propolis extract as well different extraction methods towards yield and bioactivity of the propolis extract.

Hence, this study aimed to investigate the effects of microclimate conditions on the activity of *T. laeviceps*, *T. drescheri*, and *T. biroi* cultivated in MOTIVE units in producing crude propolis. This study also aimed to investigate the effects of cultivating *T. laeviceps*, *T. drescheri*, and *T. biroi* with *P. merkusii* resin towards the productivity of crude propolis and the effects of different extraction methods on the yield, total phenolic content, total flavonoid content, and antibacterial activity of propolis extract produced by the *Tetragonula* spp.

## 2 Material and methods

### 2.1 Materials

Ethanol (PA), methanol (PA), Folin-Ciocalteu reagent, and natrium carbonates were of analytical grade obtained from Merck Co. (Darmstadt, Germany). Quercetin, gallic acid, and aluminum chloride were of analytical grade obtained from Sigma-Aldrich Chemical Co. (St.Louis, USA). Sensors used in this study includes DHT22 Sensor, Light Dependent Resistor (LDR), and TCRT5000 sensor obtained from a local sensor distributor, Ardushopid located in West Jakarta, Indonesia. Arduino UNO Microcontroller and Raspberry Pi were obtained from a local distributor, Kiosrobot located in Depok, West Java, Indonesia.

### 2.2 Cultivation of *T. laeviceps*, *T. biroi*, and *T. drescheri*

**Fig. 1:**
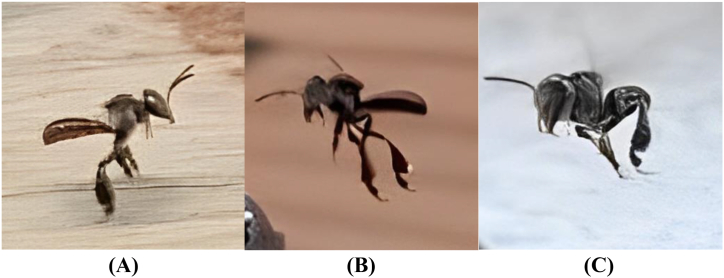
*T. laeviceps* (A)*, T. biroi* (B), *T. drescheri* (C).

**Fig. 2:**
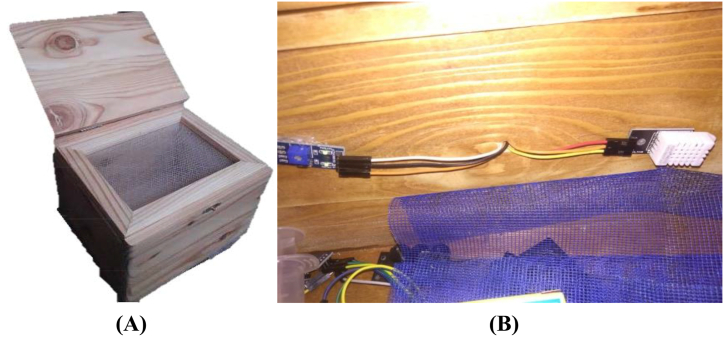
Modular *Tetragonula* Hive used for the cultivation of *T. laeviceps, T. biroi* and *T. drescheri* (A)*,* humidity and light intensity sensor installed in the Modular *Tetragonula* Hive (B).

Three different species of *Tetraogonula* spp investigated in this study particularly *T. laeviceps*, *T. biroi*, and *T. drescheri* (Fig. 1), obtained from Djikan Store, a local stingless bee supplier in Ngajuk, Pasuruan, East Java with geographical coordinates of −7.700138, 111.871215. The bees are native to the forests surrounding the bee farm and identification of the bee species was carried out by local bee farmers who possess knowledge regarding the morphology of each bee species. The bees were then cultivated at PPM Nurul Hakim, Jalan Raya Jatinangor No.138, Cikeruh, Jatinangor (geographical coordinates: -6.934323, 107.770872). The bees were acclimatized for a week before cultivation. The bees were cultivated in the MOTIVE units as shown in Fig. 2 for 1 month. The MOTIVE units used in this study were made of pine wood, with dimensions of 32.5 × 10 × 10 cm for *T. laeviceps*, 21 × 11 × 10 cm for *T. biroi*, and 32.5 × 12 × 10 cm for *T. drescheri*. The cultivation area was surrounded by *Helianthus annuus* L and *Xanthostemon* sp.*,* which serve as nectar sources for the bees while *Pinus merkusii* resin was placed in small containers around the hives. Every 2 weeks, the propolis-filled mesh was replaced with an empty mesh. During cultivation, the microclimate data and bee activity, measured in terms of the number of bees entering and leaving the hive per hour, were recorded using an Arduino UNO and a Raspberry Pi.

### 2.3 Installation of sensors and instrumentation systems in MOTIVE

The temperature and humidity of the environment within the MOTIVE units were measured using a DHT22 sensor, while light intensity data was measured using an LDR sensor. Bee activity was measured using a TCRT5000 sensor. Each of these sensors was then connected to an Arduino UNO microcontroller. The data measured by each sensor were recorded and stored in a Raspberry Pi in the form of a comma-separated values (CSV) file.

### 2.4 Harvesting of crude propolis

The crude propolis attached to the nylon mesh was separated using liquid nitrogen. The propolis productivity of each beehive was calculated using Equation (1).

[1] $$Propolis\;\left(\frac{g}{week}\right)=\frac{mass\;of\;propolis\;\left(g\right)}{2\;weeks}$$

### 2.5 Extraction of propolis

Extraction of propolis was carried out using three different methods, particularly maceration, Soxhlet, and reflux extraction. For maceration, propolis extraction was carried out using 80% ethanol solvent with a propolis: solvent ratio of 1:15 on a weight to volume (w/v) basis as suggested by Machado et al. [12]. The extraction process was carried out in an incubator shaker operating at a speed of 500 rpm at 40 °C for 2 h. As for Soxhlet extraction, the propolis was extracted based on the method described by Zin et al. [13]. Approximately 1 g of crude propolis was weighed out, wrapped in Whatman filter paper No. 2, and tied with mattress straps before being placed in the extraction chamber. 75 mL of 80% ethanol was put into a boiling flask. The Soxhlet extraction was carried out for 2 h at 85 °C. Meanwhile, for reflux extraction, approximately 1 g of the propolis was weighed out and placed into a round bottom flask. 80% ethanol was added until a propolis: ethanol ratio of 1:15 (w/v) was reached. The reflux process was carried out at 70 °C for 2 h as suggested by Cottica et al. [14]. After extraction, the propolis extract obtained from all methods were filtered with Whatman filter paper No. 20 and then stored in a dark room for further analysis.

### 2.6 Determination of propolis yield

Approximately 0.5 mL of the propolis extract that had been filtered was evaporated at 45 °C until all the solvent had fully evaporated. The remaining propolis residue was then weighed. The propolis yield can then be calculated using Equation (2).

[2] $$Yield\;\left(\%\right)=\left(\frac{m_{dry\;extract}}{v_{evaporated}}\right)\times \left(\frac{m_{supernatant}}{m_{propolis}}\right)\times 100\%$$

With m_dry extract_ is the mass of the evaporated propolis extract (g), m_propolis_ is the mass of propolis used in the extraction (g), v_evaporated_ is the volume of the filtrate used for evaporation (mL), v_supernatant_ is the volume of the supernatant obtained from filtration (mL).

### 2.7 Determination of pH and density of propolis extract

Values of pH for the propolis extract were determined using a digital pH meter whereas densities of the propolis extract were determined using a pycnometer.

### 2.8 Determination of phenolic content

Phenolic content of the samples was determined following the method described in Machado et al. [12]. A standard curve was prepared by dissolving 0.01 g of gallic acid in 10 mL of 80% ethanol to obtain a standard solution of gallic acid with a concentration of 1000 g/mL. The standard gallic acid solution was then diluted using serial dilution to obtain gallic acid solutions with concentrations of 500 g/mL, 250 g/mL, 175 g/mL, 100 g/ml, and 50 g/mL. In addition, an 80% ethanol solution was prepared as a blank. 3.75 g of solid Na_2_CO_3_ was dissolved in 50 mL of distilled water to obtain a 7.5% (w/v) Na_2_CO_3_ solution. 0.5 mL of each gallic acid solution, as well as the blank, was allowed to react with 2 mL of Folin-Ciocalteu reagent and 2.5 mL of 7.5% Na_2_CO_3_ solution. The solution was incubated for 5 min in a water bath at 50 °C. The absorbance of each gallic acid solution and the blank was measured at a wavelength of 765 nm using a UV–Vis spectrophotometer. The absorbance values obtained were used to plot a calibration curve; the equation of the line in the curve was used to calculate the phenolic content of propolis. To calculate the phenolic content of the propolis samples, the supernatant extracted from propolis was evaporated, and the residue was dissolved in 5 mL of 80% ethanol before being allowed to react with 2 mL of Folin-Ciocalteu reagent and 2.5 mL of 7.5% Na_2_CO_3_. The solution was then incubated for 5 min in a water bath at 50 °C, and its absorbance at 765 nm was measured as described in the preparation of the standard solutions. Similar procedures were carried out to determine the phenolic content of *P. merkusii* resin. The phenolic content in the sample solution was considered as the equivalent level of gallic acid with units of microgram gallic acid equivalent (μg GAE) and was determined using Equation (5).

[5] $$Phenolic\;content\;\left(mg\;GAE/mL\right)=\left(\frac{A-0.0223}{0.00528}\right)\times 1000\;\mu g/1\;mg$$

The values of 0.0223 and 0.00528 are constant and coefficient of the gallic acid calibration curve equation, and A is the absorbance of the sample. The phenolic content in the whole propolis extract can be determined by Equation (6).

[6] $$Phenolic\;content\;\left(mg\;GAE/g\right)=\frac{Phenolic\;content\;\left(mg\frac{GAE}{ml}\right)}{m_{extract}}\times V_{sol}$$

With m_extract_ was the mass of the evaporated propolis extract. V_v__=__sol_ was the volume of propolis extract mixed with ethanol (mL).

### 2.9 Determination of flavonoid content

Flavonoid content of the samples was determined according to the method described by Machado et al. [12]. A standard curve was prepared by dissolving 0.01 g of quercetin in 10 mL of 80% methanol to obtain a standard solution of quercetin with a concentration of 1000 g/mL. The quercetin standard solution was then diluted using serial dilution to obtain quercetin solutions with concentrations of 100 g/mL, 50 g/mL, 25 g/mL, 12.5 g/ml, and 6.25 g/mL. In addition, an 80% methanol solution was prepared as a blank. Approximately 2% (w/v) AlCl_3_ solution was obtained by dissolving 0.001 g of solid AlCl_3_ in 50 mL of 80% methanol. Around 2 mL of each quercetin solution, as well as the blank, were allowed to react with 2 mL of the 2% AlCl_3_ solution and incubated for 30 min. The absorbance of each solution was measured at a wavelength of 415 nm using a UV–Vis spectrophotometer. The obtained absorbance values were then used to plot a calibration curve; the equation of the line in the curve was used to calculate the flavonoid content of the propolis extract. To calculate the flavonoid content of a propolis sample, the solvent presence in the propolis extract was evaporated, and the residue was dissolved in 10 mL of 80% methanol and allowed to react with 2 mL of 2% AlCl_3_ solution. The solution was then incubated for 30 min, and its absorbance at 415 nm was measured as described above. Similar procedures were carried out to determine the flavonoid content of *P. merkusii* resin. The flavonoid content in the sample was considered as the equivalent level of quercetin in micrograms of quercetin equivalent (μg QE) and was determined using Equation (3).

[3] $$Flavonoid\;content\;\left(mg\frac{QE}{mL}\right)=\left(\frac{A+0.037}{0.0276}\right)\times 1000\;\mu g/1\;mg$$

The values of 0.037 and 0.0276 are the constants and coefficients of the quercetin calibration curve equation, and A is the absorbance of the sample. The values of 0.037 and 0.0276 are the constants and coefficients of the quercetin calibration curve equation, and A is the absorbance of the sample. The flavonoid content in the whole propolis extract can be determined by Equation (4).

[4] $$Flavonoid\;content\;\left(mg\frac{QE}{g}\right)=\;\left(\frac{Flavonoid\;content\;\left(mg\;QE/mL\right)\;x\;V_{sol}}{m_{extract}}\right)$$

With _mextract_ is the mass of the propolis extract (g), V_sol_ is the volume of propolis extract mixed with methanol (mL).

### 2.10 Determination of antibacterial activity using a disc diffusion method

The antibacterial properties of the propolis extract were analyzed using a disc diffusion method, also known as the Kirby-Bauer test [[15], [16], [17]]. This antimicrobial test was carried out at the Central Laboratory, Padjadjaran University, Jatinangor, Sumedang. Approximately 38 g of Mueller Hinton Agar (MHA) medium powder was dissolved in 1 L of distilled water to produce an MHA medium. The solution was heated to dissolve the medium and sterilized in an autoclave at a temperature of 121 °C for 15 min before being cooled to 45–50 °C and homogenized. Following these preparatory steps, up to 10 mL of the media was transferred into a sterilized Petri dish and left to solidify. When the agar had formed, *E. coli* and *S. aureus* bacteria were inserted using a sterile cotton stick and then incubated in an incubator at 37 °C for 24 h. Disc paper with a diameter of 6 mm was dipped into the sample extract of propolis, 10 ppm amoxicillin (positive control), and 80% ethanol (negative control). The filter paper was then placed into the Petri dish containing the *S. aureus* culture and incubated at 37 °C for 24 h. A bright zone was observed to have formed around the paper disc; this was measured using a ruler and the help of a magnifying glass to determine the effectiveness of the propolis extract in inhibiting bacterial growth [18].

### 2.11 Statistical analysis

All the analysis were performed in triplicate and expressed as mean ± standard deviation. Differences were tested by analysis of variance (ANOVA) using Minitab version 21.1.0. A significance level of P < 0.05 was used. Spearman tests were also performed using Minitab version 21.1.0 to evaluate the correlation between activity of bees and microclimate variables. This analysis was also carried out using the Minitab version 21.1.0.

## 3 Results and discussions

### 3.1 Effects of microclimate conditions on bee activity and propolis productivity

**Fig. 3:**
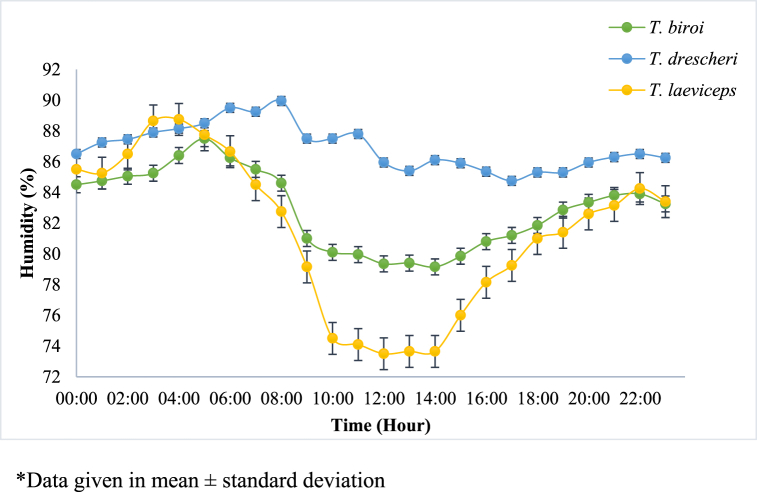
Daily relative humidity profile of different bee species at Jatinangor. *Data given in mean ± standard deviation.

**Fig. 4:**
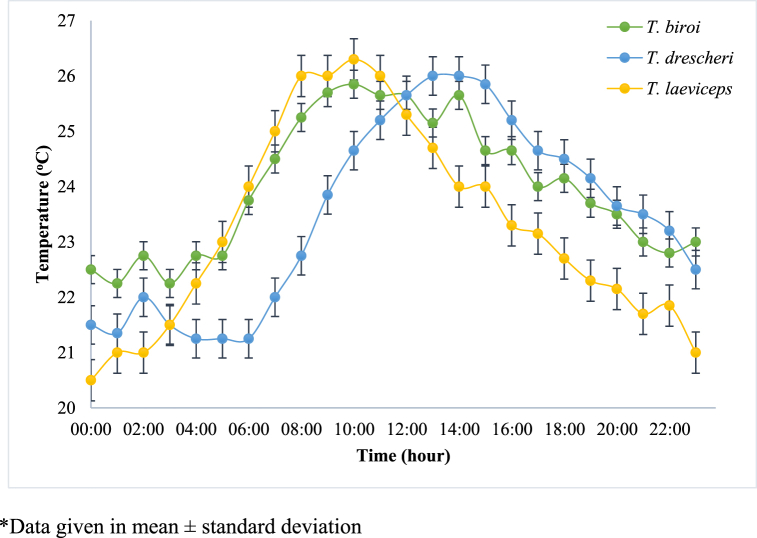
Daily temperature profile of different bee species at Jatinangor. *Data given in mean ± standard deviation.

**Fig. 5:**
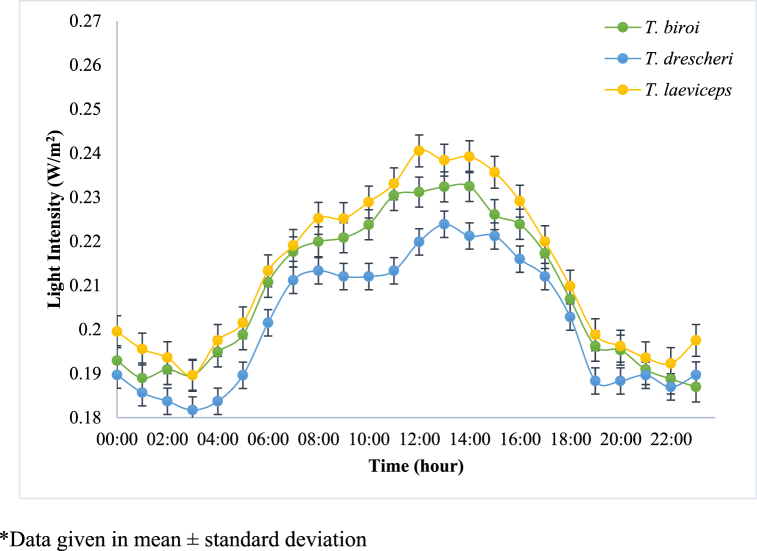
Daily light intensity profile of different bee species at Jatinangor. *Data given in mean ± standard deviation.

**Fig. 6:**
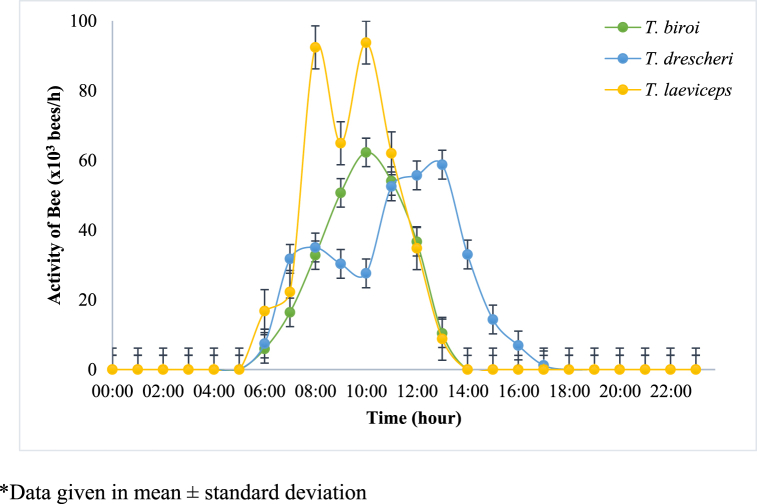
Daily activity of different bee species at Jatinangor expressed as average number of bees per hour entering and exiting the hive. *Data given in mean ± standard deviation.

In this study, sensors were installed in the MOTIVE units to monitor the microclimate conditions in the hives and the colony activity of each bee species. The daily profiles of the average humidity, temperature, and light intensity in the hive, as well as the activity of each bee species, are shown in Fig. 3, Fig. 4, Fig. 5, Fig. 6, respectively. Spearman correlation coefficients of microclimate conditions in the beehive of each bee species are shown in Table 1.

**Table 1:** Spearman correlation of microclimate conditions in beehive of each bee species.

Species	Coefficient		
	Humidity	Temperature	Light intensity
*T. laeviceps*	−0.379	0.828**	0.544*
*T. biroi*	−0.340	0.702**	0.459*
*T. drescheri*	0.030	0.678**	0.807**

* Significant correlation with P < 0.05.

** Significant correlation with P < 0.001.

The bees were cultivated at Cikeruh, Jatinangor, Sumedang Regency, Indonesia. The average relative humidity and precipitation in Cikeruh during the cultivation period were 83% and 393 mm, while the average low and high temperatures were 21.3 °C and 30 °C, respectively [19]. The relative humidity in each beehive was found to be at its highest in the morning; it decreased during the day but increased again in the afternoon (Fig. 3). The range of humidity in the hives of *T. drescheri*, *T. biroi*, and *T. laeviceps* ranged from 85.3 to 90.0%, 79.2–86.4%, and 73.7–88.8%, respectively. The humidity of the hive is known to affect bee activity: the greater the humidity within the hive, the lower the bee activity [20]. According to Salatnaya et al. [21], bee activity is greatest when the humidity of the hive falls between 55 and 71%. The relative humidity of the hive and the surrounding environment was higher than optimal which affect the activity of the bees. However, based on the spearman test, there was no significant correlation between relative humidity and bee activity for all beehives (P-value > 0.05, Table 1).

The temperature in the hive for each bee species exhibited the same trend (Fig. 4). The temperature in the beehives was low in the morning and increased over the course of the day before decreasing again in the afternoon. The temperature ranges in the hives of *T. drescheri*, *T. biroi*, and *T. laeviceps* ranged from 21.3 to 26.2 °C, 22.3–25.9 °C, and 20.5–26.3 °C, respectively. According to Salatnaya et al. [21], the optimum temperature range for beehives falls between 26 and 28 °C. Bees insulate their hives from external temperature fluctuations using propolis, allowing them to maintain the optimal temperature conditions within the hive. These temperature conditions are required to ensure that each bee’s metabolic processes remain optimized. The temperature ranges of the beehives monitored in this study sometimes fell below the optimal conditions for the bees. In particular, the *T. laeviceps* hives had the lowest average temperature compared to the hives of other bee species; this is consistent with their lower propolis productivity compared to the other species. Based on the spearman test, there was a positive significant correlation between temperature and the activity of bees for all beehives (P-value < 0.001, Table 1). According to Boontop et al. [20], increasing environmental temperature is correlated with an increase in bee activity, measured in terms of the number of bees entering and leaving the hive. Jager et al. [22], suggests that increasing temperature causes resins and plant waxes to soften, allowing bees to process them. On very hot days, bees start work early in the morning; in contrast, bees will not collect resin at low temperatures. Figs. 4 and 6 show that bee activity was highest at high temperatures.

Fig. 5 shows that the light intensity in the beehive exhibited the same patterns as the temperature variations. The light intensity in the hives was low in the mornings, increased over the course of the day, and decreased again in the afternoon. The range of natural light intensity in the hives of *T. drescheri*, *T. biroi*, and *T. laeviceps* were 0.182–0.224 W/m^2^, 0.187–0.232 W/m^2^, and 0.190–0.241 W/m^2^, respectively. Salatnaya et al. [21] found that bee activity was highest when the natural light intensity was between 370 and 721 W/m^2^. In this study, the light intensity in the hives was found to be very low. Although light entering the hive can cause the bees to cover these gaps with propolis, low light intensities can maintain the health of the bee colony, allowing the bees to produce propolis at an optimal rate [23]. The light intensities recorded in the hives of each species did not vary significantly in this study, suggesting that it was not the primary cause of the differences in propolis productivity. However, based on the spearman test, there was a positive significant correlation between light intensity of all beehives and the activity of bees (P-value < 0.05, Table 1).

Propolis productivity was found to be closely related to total bee activity. In this study, the bees produced propolis from the resin of *P. merkusii* which was placed about 1 m from the entrance of the hive. Fig. 6 shows that the activity of *T. drescheri* lasted the longest; these bees were active from 06.00 to 17.00. Although *T. laeviceps* activity also began at 06.00, their activity began to decrease at around 14.00 whereas for *T. biroi* their activity began at 06.00 but their activity started to decrease sooner at around 10.00 as compared to the other species. Hence, the long duration of *T. drescheri* activity is consistent with their high propolis productivity. Nevertheless, further studies need to be carried out to better understand the different profiles exhibited by each bee species.

### 3.2 Propolis productivity

*T. drescheri* and *T. laeviceps* exhibited the highest and lowest propolis productivity*,* respectively. The average propolis productivity of each bee species investigated in this study was compared with productivities observed in previous studies (Table 2). It was observed that propolis productivity was proportional to the body size of the bee species; *T. drescheri* was the largest species in this study, followed by *T. biroi* and *T. laeviceps*. The morphometric characteristics of each bee species in this study are described in Table 3. Statistical analysis indicated significant differences (p < 0.001) between species for all characteristics.

**Table 2:** Average propolis productivity of *T. laeviceps*, *T. biroi* and *T. drescheri* cultivated at Jatinangor.

Species	Hive dimension	Productivity (g/coloni/week)	Ratio of propolis productivity with hive area (mg/cm^2^)	Source
*T.laeviceps*	21 × 14 × 18 cm^3^	0.95–1.25	3.23–4.25	[5]
	21 × 18 × 14 cm^3^	0.49–3.72	1/30–9.84	[24]
	26 × 22 × 17 cm^3^	2.25–6.75	3.93–11.80	[25]
	30 × 26 × 17 cm^3^	3.97–6.15	5.08–7.88	[26]
	32.5 × 10 × 10 cm^3^	1.22–3.19	5.11–8.18	This study
*T.biroi*	21 × 11 × 10 cm^3^	1.72–2.08	7.45–9.00	This study
*T.drescheri*	32.5 × 10 × 12 cm^3^	2.31–5.88	7.11–9.05	This study

*Data given in mean ± standard deviation.

**Table 3:** Morphometric of *T. laeviceps*, *T. biroi* and *T. drescheri*.

Characteristics	Morphometric (mm)		
	T.laeviceps	T.biroi	T.drescheri
Body length	3.42 ± 0.02	3.94 ± 0.02	4.64 ± 0.01
Hind tibia width	0.47 ± 0.02	0.54 ± 0.02	0.74 ± 0.01
Hind tibia length	1.41 ± 0.02	1.59 ± 0.02	2.15 ± 0.01

*Data given in mean ± standard deviation.

The minimum value of propolis productivity per unit area of beehive recorded in this study was greater than that of previous studies. This could be due to the availability of *P. merkusii* resin at the cultivation site, which was provided to the bees by placing *P. merkusii* resin in a pot located about 1 m from the entrance of the hives. The availability of feed in the form of resin is one of the factors that affect the production of propolis [27]. Bees carry this resin by using the tibia of the bee's hind legs, commonly called the pollen basket. These areas contain fine hairs that can hold resin and allow pollen to stay attached. The body size of each bee species controls the bee's ability to carry different amounts of resin: larger bees can carry more resin than smaller bees.

The difference in propolis productivity in this study could also be due to the competition between colonies with regard to the collection of the pine resin. Although each hive was provided with pine resin, it was observed that bee colonies often collected pine resin from the hives of other colonies. Frequent agonistic behaviour between bees of different species was also observed. Agonistic behaviour refers to aggressive behaviour, including fighting, threatening, and showing off their strength to mark an organism’s territory and access to food sources [28]. Aggressive encounters between stingless bee species significantly reduce the amount of time that the bees spend searching for additional food sources; this causes the bees to collect less food per visit [29].

In this study, it was observed that the other bee species were threatened by the presence of *T. drescheri*, the largest of the bee species. *T. laeviceps*, which had the smallest body size among the bees in this study, were often observed to be flying away from the pine resin that had been provided as they felt threatened by the presence of *T. drescheri*. This behaviour caused *T. laeviceps* to have less time to collect resin and could be an explanation for why *T. laeviceps* had the lowest average propolis productivity.

In addition to external factors in the form of feed availability and competition between bee colonies, propolis productivity is also influenced by internal factors, including the different needs of each colony and the number of colonies in the hive [30]. External factors such as changes in the microclimate in the hive and the surrounding environment also affect bees in producing propolis [21].

### 3.3 Physiochemical properties, yield and composition of propolis extract

The calculated density and pH of the propolis extracts obtained in this study are presented in Table 4 whereas the calculated yield of propolis extracts obtained in this study are compared with the literature as shown in Table 5. Based on the ANOVA, the pH (P-value < 0.001) and density (P-value < 0.05) of propolis extract (Table 4) is significantly different among different extracting method (P-value < 0.01) and significantly different among different bee species (P-value < 0.01). The pH of propolis is influenced by the content of organic and inorganic acids. The acidity level of propolis can help propolis’s resistance and stability to microorganisms. Meanwhile, the determination of density aims to describe the chemical content dissolved in the extract. The density of the propolis extracts of all samples was in the range of 0.86–0.86 g/mL that resembles the previous results reported by Syukri et al. [31].

**Table 4:** Density and pH of propolis extract produced by *T. laeviceps, T. biroi*, and *T. drescheri*.

Species	Density	pH
T. laeviceps	0.87 ± 0.001	5.00 ± 0.006
T. biroi	0.87 ± 0.001	4.85 ± 0.006
T. drescheri	0.86 ± 0.001	5.30 ± 0.006

*Data given in mean ± standard deviation.

**Table 5:** Yield of propolis extract produced by *T. laeviceps*, *T. biroi* and *T. drescheri*.

Species	Extraction Method	Solvent	Propolis/Solvent (%w/v)	Temperature (°C)	Time (h)	% Yield
*T. laeviceps*	Maceration	Ethanol 80%	6.6	40	2	15.12 ± 1.23
		Ethanol 95%	22.5	15	20	20 [32]
	Reflux	Ethanol 80%	6.6	70	2	14.03 ± 1.79
	Soxhlet	Ethanol 80%	0.6	85	2	22.90 ± 1.11
*T.biroi*	Maceration	Ethanol 80%	6.6	40	2	20.32 ± 4.83
		Ethanol 70%	60	25	24	10.01 ± 0.16 [33]
	Reflux	Ethanol 80%	6.6	70	2	13.38 ± 1.47
	Soxhlet	Ethanol 80%	0.6	85	2	20.41 ± 0.23
*T. drescheri*	Maceration	Ethanol 80%	6.6	40	2	24.17 ± 5.99
	Reflux	Ethanol 80%	6.6	70	2	19.30 ± 5.84
	Soxhlet	Ethanol 80%	0.6	85	2	32.45 ± 0.90

*Data given in mean ± standard deviation.

The yield of the propolis extract from each bee species varied significantly. Based on the ANOVA, the yield of propolis extract is significantly different among different extracting methods (P-value < 0.01) and significantly different among different bee species (P-value < 0.01). The highest yield observed in this study was from *T. drescheri*, while the lowest was from *T. laeviceps*. The difference in yields could be influenced by the solubility and polarity of the extracted compounds. In addition, the choice of solvent in the extraction greatly affects the yield of active substances extracted. The yield of active substances can be increased by increasing the compatibility between the polarity of the solvent and the active substances within the raw material. The fact that different yields were obtained from the propolis of each stingless bee species indicates that the properties of these propolis samples were different. In addition, the extraction method can also affect the number of compounds extracted, while the concentration of ethanol affects the type of compounds extracted [14]. The properties of the solvent, the temperature and duration of the extraction process, and the composition and physical characteristics of propolis samples can all greatly affect the yield and quality of the final extracts [12].

In this study, the greatest yield was obtained from Soxhlet extraction compared to the maceration and reflux methods. Soxhlet extraction is more effective than maceration in terms of obtaining greater yields from propolis extracts [6]. This could be due to the greater volume of solvents used in Soxhlet extraction. The ratio of crude propolis to solvent for maceration and reflux extraction was 1:15 whereas for Soxhlet extraction, a higher crude propolis to solvent ratio of 1:150 was applied during the study to allow condensation of the solvent for at least 1 cycle. The use of more solvents in the extraction process can cause the solubility of substances in propolis to increase and hence allowing greater yields for the Soxhlet extraction.

In addition, the Soxhlet extraction was carried out at a higher temperature (85 °C) as compared to the maceration (40 °C) and reflux (70 °C) method. Higher temperatures during the extraction process can increase the diffusion coefficient and solubility of the active substances [34]. Although high temperatures were also used in the reflux method, the propolis was not directly heated during Soxhlet extraction. During Soxhlet extraction, any evaporated solvent was immediately condensed and returned to the flask, submerging the propolis for the entire duration of the extraction process. In the reflux method, both the solvent and propolis were heated directly; hence, some compounds in the propolis extract may have been damaged during the process.

The stirring step implemented in the maceration method caused it to obtain higher yields than the reflux method. Stirring causes the solubility of the active substance in the solvent to increase, which consequently increases the mass transfer rate of the active substance from the raw material to the solvent. Stirring can also prevent the agglomeration of the active substance in the solvent, which may cause the substances to precipitate [35].

### 3.4 Bioactivities of propolis extract

The concentration of bioactive compounds particularly flavonoids and phenolics of the propolis extract obtained using different extraction methods for each bee species are compared with the literature and the results are shown in Table 6. Statistically, the total phenolic content of propolis extract is significantly different among different extraction methods (P-value < 0.05) and significantly different among different bee species (P-value < 0.01). The total flavonoid content of propolis is significantly different among different bee species (P-value < 0.001) but not significantly different among different extraction methods (P-value > 0.05).

**Table 6:** Total phenolic and flavonoid content of propolis extract.

Species	Methods	Total Phenolic Content (mg GAE/g)	Total Flavonoid Content (mg QE/g)
*T.laeviceps*^*b*^	Maceration	343.93 ± 44.32^*b*^	35.77 ± 9.94^*a*^
	Reflux	270.97 ± 119.3^*a,b*^	30.58 ± 5.53 ^*a*^
	Soxhlet	136.48 ± 13.90^*a*^	20.12 ± 0.95 ^*a*^
*T.biroi*^*a*^	Maceration	123.81 ± 24.69 ^*b*^	5.48 ± 3.31 ^*a*^
	Reflux	98.13 ± 48.82 ^*a,b*^	4.41 ± 0.55^*a*^
	Soxhlet	104.09 ± 3.86 ^*a*^	5.10 ± 0.04 ^*a*^
*T.drescheri*^*a*^	Maceration	225.87 ± 70.36^*b*^	14.57 ± 9.30 ^*a*^
	Reflux	170.22 ± 70.34^*a,b*^	12.11 ± 3.83 ^*a*^
	Soxhlet	117.15 ± 2.34^*a*^	7.93 ± 0.19 ^*a*^

*Data given in mean ± standard deviation. Different letters in the species column indicate statistically significant differences between species. Different letters in the phenolic and flavonoid columns indicate statistical differences between extraction methods (P-value <0.05, Duncan's test).

The phenolic content and flavonoid content of propolis extract investigated in this study are higher than the phenolic content and flavonoid content of *P. merkusii* resin used in this study with average values of 64.72 ± 3.65 mg GAE/g and 0.44 ± 0.01 mg QE/g, respectively. The data in Table 7 also shows that the propolis extract investigated in this study has higher values as compared to the findings reported by Fikri et al. [7]. Propolis from *T. laeviceps* extracted by maceration, Soxhlet, and reflux methods had the highest flavonoid and phenolic content, while propolis from *T. biroi* had the lowest flavonoid and phenolic content. These were contrary to the results obtained by Fikri et al. [7] in which propolis produced by *T. biroi* had the highest flavonoid and phenolic content. These differences could be due to the differences in the source of the propolis; in this study, the bees were provided with *P. merkusii* resin, while the bees in Fikri et al. [7] obtained propolis from various types of plants. The flavonoid and phenolic content of propolis is known to be influenced by the source of the resin, the time and age of the resin during extraction, and its phytogeographic location [36].

**Table 7:** Antibacterial activity of propolis extract against *E. coli* and *S.aureus*.

Sample	Extraction Method	Concentration	Inhibition Diameter (mm)	
			*E. coli*	*S. aureus*
*T.drescheri*	Maceration	66.7 mg/mL	6.00	9.70 ± 0.7
	Soxhlet	6.7 mg/mL	6.00	8.75 ± 0.49
	Reflux	66.67 mg/mL	6.00	6.83 ± 0.32
*T. laeviceps*	Soxhlet	6.7 mg/mL	6.00	7.70 ± 0.14
*T. biroi*	Soxhlet	6.7 mg/mL	6.00	6.58 ± 0.04
Resin *P.merkusii*	–	100%	6.5 ± 0.1	7.45 ± 0.21
Amoxicillin	–	0.1 mg/mL	13.55 ± 0.05	23.50 ± 0.28
Ethanol	–	80%	6.00	6.00

**Fig. 7:**
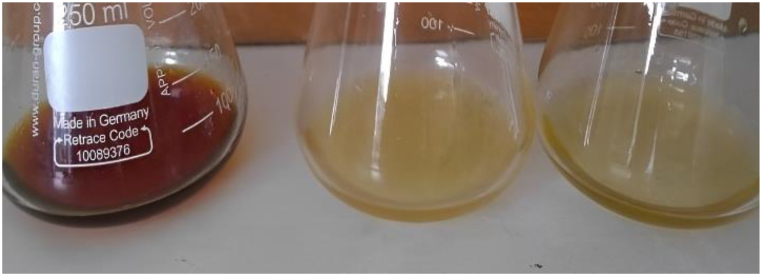
Samples of propolis extract from *T. laeviceps* (left), *T. biroi* (centre), *T. drescheri* (right).

The propolis extracts produced by *T. laeviceps* had a dark brown colour, while the *T. drescheri* propolis extract had a brown colour, and the *T. biroi* propolis extract had the lightest shade of brown (as shown in Fig. 7). The darker the colour of the propolis extract, the greater the flavonoid content [37]. The results of this study were consistent with those found in the literature. There are differences in the chemical composition of the propolis extracts from each bee species, especially in terms of their flavonoid and phenolic content, due to the genetic variability of bees [38]. The flavonoids and phenolics are secondary metabolites produced by organisms as a self-defence mechanism. In this study, *T. laeviceps* was found to be intimidated by the other bee species due to its small body size. As a form of defence, *T. laeviceps* produced higher levels of flavonoid and phenolic compounds than the other species. Flavonoid and phenolic compounds in propolis are known to have antioxidant and antibacterial properties, providing protection from predators, parasites, and pathogens, and maintaining a healthy environment for the bees [39].

The propolis extracts were also shown to have antibacterial properties as shown in Table 7. According to Davis & Stout [40], the inhibition diameter for antimicrobial activity can be classified as very strong (inhibition diameter ≥ 20 mm), strong (10 mm ≤ inhibition diameter < 20 mm), moderate (5 mm ≤ inhibition diameter < 10 mm), and weak (inhibition diameter < 5 mm). As mentioned in the previous section, alpha-pinene was detected in the propolis extract of all species. Masruri et al. [41] showed that alpha-pinene compounds possess antibacterial properties and could form 8.3 mm and 8.9 mm inhibition zones against *S. aureus* and *E. coli*, respectively. In this study, *P. merkusii* resin was found to inhibit the growth of both *E. coli* (gram-negative bacteria) and *S. aureus* (gram-positive bacteria) with an inhibition diameter of 6.5 ± 0.1 mm and 7.45 ± 0.21 mm, respectively. The inhibition diameter of the propolis extract against *S. aureus* varies from 6.58 ± 0.04 mm to 9.70 ± 0.7 mm which be considered as moderate antimicrobial activity. However, all the propolis extract demonstrate a similar inhibition diameter with ethanol against *E. coli*. Most probably, this may be due to the outer membrane of the gram-negative bacteria, which provides it with some antibiotic resistance [42].

The *T. laeviceps* propolis extract had stronger antibacterial properties than the *T. biroi* propolis extract. In addition, tests on the propolis extract from *T. drescheri* showed that propolis extract produced by the maceration had stronger antibacterial properties than those produced by the other methods. The concentration of flavonoids in the propolis extract of *T. laeviceps* was found to be higher than *T. Biroi*. In addition, the extract acquired using maceration had higher flavonoid contents than those obtained by other methods. High flavonoid concentrations can contribute to the antibacterial properties of the propolis extract: one of the propolis compounds that was found to have antibacterial properties was a flavonoid group compound [43].

Previous research demonstrated that a 20 mg/mL of propolis extract derived from *T. laeviceps* exhibited antibacterial activity, creating an inhibition zone around *S. aureus* with a diameter of 0.67 ± 0.05 mm [44]. In another study, a propolis extract from *T. biroi* did not exhibit any antibacterial activity with respect to *S. aureus* [45]*.* The differences in the antibacterial properties observed between the previous study and the results of this study suggest that the antibacterial nature of propolis does not depend on the type of bee species but on the chemical composition of the propolis, which is influenced by the geographical location of the beehives, as this affects the types of vegetation that are available as resin sources [36].

In this study, the bees used the *P. merkusii* resin provided to produce propolis. Wijayati et al. [46] showed that *P. merkusii* resin exhibited antibacterial properties, allowing for the formation of inhibition zones of 2.2–13.8 mm and 2.37–9.13 mm against *S. aureus* and *E. coli*, respectively. In contrast, Tillah et al. [47] found that *P. merkusii* resin produced an inhibition zone of 7.71 mm against *S. aureus*, but did not exhibit any antibacterial properties with respect to *E. coli*. One factor that affects the potential of an antibacterial substance is the concentration of bacterial suspension; higher concentrations of cells can affect a substance’s antibacterial properties [48]. These properties can also be influenced by the age, type, and state of the bacteria [46].

## 4 Conclusions

The effects of microclimate conditions on the activities of *Tetragonula* spp. have been determined. Based on the statistical analysis, there was a positive significant correlation between temperature and light intensity towards the activity *Tetragonula* spp. exiting and entering the beehives*.* The influence of cultivating *Tetragonula* spp. with resin from *Pinus merkusii* on the productivity of crude propolis and composition of propolis extract as well different extraction methods towards yield and bioactivity of the propolis extract also have been determined. The productivity of crude propolis lies in the range of 1.22–5.88 g/colony/week whereas the yield of propolis extract varies from 15.12 to 24.17%. The phenolic and flavonoid content of the propolis extract is in range of 123.81–343.93 mg GAE/g and 5.48–35.77 mg QE/g, respectively. The highest propolis yield (32.45 ± 0.90%) was obtained from the crude propolis produced by *T. drescheri* followed by Soxhlet extraction method. Propolis extract with the highest phenolic content (343.93 ± 44.32 mg GAE/g) and flavonoid content (35.77 ± 9.94 mg QE/g) was obtained from the propolis produced by *T. laeviceps* followed by maceration method. All the propolis extracts inhibited the growth of *Staphylococcus aureus* with moderate antimicrobial activity.

## Author contribution statement

Muhammad Yusuf Abduh: Conceived and designed the experiments; Analyzed and interpreted the data; Contributed reagents, materials, analysis tools or data; Wrote the paper.

Fahmi Ramdhani; Albert Setiawan; Ghiffry Rifqialdi: Performed the experiments; Analyzed and interpreted the data; Wrote the paper.

Ima Mulayama Zainudin: Conceived and designed the experiments; Analyzed and interpreted the data; Wrote the paper.

Anasya Rahmawati: Analyzed and interpreted the data; Wrote the paper.

## Data availability statement

Data will be made available on request.

## Declaration of competing interest

The authors declare that they have no known competing financial interests or personal relationships that could have appeared to influence the work reported in this paper.
